# Fast Food Pattern and Cardiometabolic Disorders: A Review of Current Studies

**DOI:** 10.15171/hpp.2015.028

**Published:** 2016-01-30

**Authors:** Zahra Bahadoran, Parvin Mirmiran, Fereidoun Azizi

**Affiliations:** ^1^Nutrition and Endocrine Research Center, Research Institute for Endocrine Sciences, Shahid Beheshti University of Medical Sciences, Tehran, Iran; ^2^Endocrine Research Center, Research Institute for Endocrine Sciences, Shahid Beheshti University of Medical Sciences, Tehran, Iran

**Keywords:** Fast food, Obesity, Metabolic syndrome, Insulin resistance, Cardiovascular disease, Diabetes

## Abstract

**
Background:** There are growing concern globally regarding the alarming trend of fast food consumption and its related cardiometabolic outcomes including overweight and obesity. This study aimed to review the current evidences available in relation to adverse effects of fast food pattern on cardiometa­bolic risk factors.

**
Methods:** Relevant articles including epidemiological and clinical studies with appropriate design and good quality were obtained through searches of the Medline, PubMed, Scopus databases and Google scholar with related key words including "fast foods", "processed foods", "obesity", "overweight", "insulin resistance", "diabetes", "cardiovascular disease", "metabolic syndrome", "dyslipidemia" and "hypertension".

**
Results:** Fast food consumption and out-of-home eating behavior is a main risk factor for lower diet quality, higher calorie and fat intake and lower micronutrients density of diet. Frequent consumption of fast foods was accompanied with overweight and abdominal fat gain, impaired insulin and glucose homeostasis, lipid and lipoprotein disorders, induction of systemic inflammation and oxidative stress. Higher fast food consumption also increases the risk of developmental diabetes, metabolic syndrome and cardiovascular disease.

**
Conclusion:** This review provides further evidence warning us against the irreparable effects of fast food consumption on public health especially the increasing global burden of obesity and cardiovascu­lar diseases.

## Introduction

A growing trend of fast food consumption along with alarming trend of cardiometabolic disorders is considered as a globally health problem. Although there is no agreement on the definition of fast food, it is mainly defined as "easily prepared processed food served in snack bars and restaurants as a quick meal or to be taken away" in dictionaries and encyclopedias; industrial foods such as canned foods or snacks may also considered as fast foods.^1^ In the recent years, an increasing globally popularity have been developed regarding the fast foods and take-away foods marketing. Out-of-home meals and fast foods are rich in highly processed meat and refined carbohydrate, sodium, total fat, saturated and trans fatty acids, cholesterol, and poor in essential nutrients and dietary fibers; ^2^ the fast food pattern also has undesirable effects on overall diet quality especially in children and adolescents.^2-4^ Fast food consumption and out-of-home eating behavior is a main risk factor for higher calorie and fat intake and lower micronutrients density of diet.^3,5^ Frequent consumption of fast foods is one of the main reasons for rising trends of overweight and obesity, cardiovascular disease, type 2 diabetes and other metabolic abnormalities.^3,6-8^ Higher availability of fast food services is associated with higher mortality and hospital admission rates for acute coronary heart disease as well as a higher risk of overweight and obesity.^9,10^

Considering to growing interest to Western dietary patterns and trend of fast food consumption along with global burden of cardiovascular diseases, diabetes, obesity and hypertension, and the lack of a comprehensive review study on cardiometabolic outcomes of these dietary patterns, this study aimed to review the current evidence in relation to adverse effects of fast food patterns on non-communicable diseases with focusing on cardiometabolic risk factors.

## Materials and Methods

This is a narrative review article. The original research articles were reviewed published in English from 1990 to 2014. To search the articles, a number of databases and search engines, including PubMed, Medline, Scopus and Google Scholar were used. The references of the articles were also reviewed to identify papers that are more relevant. Searches were conducted with the search terms “fast foods”, “processed foods”, “obesity”, “overweight”, “insulin resistance”, “diabetes”, “cardiovascular disease”, “metabolic syndrome”, “dyslipidemia” and “hypertension”. Relevant articles including both epidemiological including cohort, case-control, cross-sectional and clinical studies were assessed for initial eligibility. Studies with English language evaluated the association between fast food consumption with cardiometabolic risk factors, with appropriate design and good quality (e.g. accurate definition of exposure and outcome, study population, clearly defined statistical methods) were included.

### Ethical consideration

Ethical issues which have been considered for this study was included prevention of selective reporting bias of the papers, and honesty in reporting of the results of the studies. Moreover, related references have been carefully cited throughout the manuscript.

## Results

### Fast food consumption and the risk of overweight and obesity

The alarming trend in the acceleration of overweight and obesity is mainly attributed to changes in lifestyle determinants and environmental factors. A rapid on-going nutrition transition with progressive shift to a westernized diet, in particular higher consumption of industrial and processed foods, and sweetened beverages are major factors contributing to the global epidemic of obesity.^11^ Among various dietary factors, out-of-home eating patterns and regular consumption of fast food have been proposed as determinant factors in the prevalence of obesity and severe weight gain over time;^12,13^ an association which has been confirmed in both prospective and cross-sectional studies. In Table 1, the associations of fast food consumption with anthropometric measures and risk of obesity in cohort and cross-sectional studies were reviewed.

Frequent consumption of fast food, ≥2 times/week, compared to <1 time/week, has been accompanied with ≥4.5 kg weight gain during a fifteen-year follow-up of US adolescents and young adults.^6^

Participants of the Coronary Artery Risk Development in Young Adults (CARDIA) study who were in the highest compared to the lowest quartile of fast food consumption, had higher weight (adjusted mean=5.6 kg, 95% CI= 2.1-9.2), and waist circumference (adjusted mean=5.3 cm, 95% CI=2.8-7.9) after a 13-yrfollow-up; in this study, fast food intake was associated with 13-yrchanges in body weight (β=0.15, 95% CI= 0.06-0.24) and waist circumference (β=0.12, CI= 0.04-0.20).^7^ A3-yrfollow-up of adults also showed that increased consumption of fast foods was associated with an increase in body mass index(BMI) change (β=0.05, 95% CI=0.01-0.09); each one unit increase in fast food consumption (1 time/wk) was associated with a 0.13 increase in BMI at baseline (β= 0.13, 95% CI: 0.04-0.22) and a 0.24 increase in BMI after 3years (β=0.24, 95% CI= 0.13-0.34).^14^

Participants of the Coronary Artery Risk Development in Young Adults (CARDIA) study who were in the highest compared to the lowest quartile of fast food consumption, had higher weight (adjusted mean=5.6 kg, 95% CI= 2.1-9.2), and waist circumference (adjusted mean=5.3 cm, 95% CI=2.8-7.9) after a 13-yrfollow-up; in this study, fast food intake was associated with 13-yrchanges in body weight (β=0.15, 95% CI= 0.06-0.24) and waist circumference (β=0.12, CI= 0.04-0.20).^7^ A3-yrfollow-up of adults also showed that increased consumption of fast foods was associated with an increase in body mass index (BMI) change (β=0.05, 95% CI=0.01-0.09); each one unit increase in fast food consumption (1 time/wk) was associated with a 0.13 increase in BMI at baseline (β= 0.13, 95% CI: 0.04-0.22) and a 0.24 increase in BMI after 3 years (β=0.24, 95% CI= 0.13-0.34) .^14^

**Table 1:** The association of fast food consumption with anthropometric measures and the risk of obesity in cohort and cross-sectional studies

Author	Design, study population and sample size	Findings
	Fifteen-year follow-up of US adolescents and young adults, n= 3031	Consumption of fast food, ≥2 times/week, compared to <1 time/week was accompanied with 4.5 kg more weight gain
Pereira et al., 2005 ^(6)^	Thirteen-year follow-up of young adults participated in CARDIA study, n= 3643	Highest compared to the lowest quartile of fast food consumption was accompanied with higher weight and waist circumference
Duffey et al., 2009 ^(7)^	Three-year follow-up of adults, n=3394	Increased consumption of fast foods (>1 time/wk) increased body mass index.
Duffey et al., 2007 ^(14)^	Two-year follow-up of adults participants in Mediterranean cohort study, n= 7194	More consumption of hamburger, pizza, and sausages increased risk of weight gain (≥3 kg during a 5 past year) (OR=1.2, 95% CI=1-1.4)
Bes-Rastrollo et al., 2006 ^(15)^	Cross-sectional study of school children, n=1033	Higher consumption of fast food was associated with higher BMI Z score (β=0.08, 95% CI=0.03-0.14), higher body fat (β=2.06, 95% CI=1.33-2.79) and an increased risk of obesity (OR=1.23, 95% CI=1.02-1.49).
Jeffery et al., 2006 ^(17)^	Cross-sectional study of Singaporean adults, n=1627	The risk of abdominal obesity was 1.24 (95% CI=1.03- 1.51) and 1.52 (95 % CI= 1.32- 1.77) in regular consumers and occasional consumers of fast foods.
Whitton et al., 2013 ^(19)^	A cross-sectional study of adults participated in Michigan Behavioral Risk Factor Survey	Increased risk (OR=1.81, 95% CI=1.35-2.44) of obesity was observed in adults with consuming ≥3 times/week compared to <1 time/week fast foods.
Anderson et al., 2011 ^(20)^	A cross-sectional study of Iranian men and women participated in Tehran Lipid and Glucose Study, n=1944	A significant association was observed between fast food intake and BMI (β=0.104, P<0.01) as well as waist circumference (β=0.083, P<0.01).
Bahadoran et al., 2012 ^(2)^	A cross-sectional survey on adults resident in Michigan, n=1345	A significant association was found between local concentrations of fast food outlets with body mass index (β=3.21, P<0.001) and poor diet quality (β=2.67, P<0.008).

In a Mediterranean cohort study, a higher risk of weight gain (≥3 kg during a 5 past year) (OR=1.2, 95% CI=1-1.4) was observed in adults who consumed more hamburger, pizza, and sausages; a significantly greater weight gain during a 2-year follow-up was also observed in the highest compared to the lowest quintile of fast food consumption (0.77 kg *vs.* 0.47 kg).^15^ A three-year follow-up of women also indicated that increased consumption of one fast food meal per week led to a 0.72 kg more weight gain.^21^

Cross-sectional studies^2,16,20-22^ also reported a positive association between consumption of fast food and the anthropometric measures in different populations and various age-groups; in school children, consumption of fast food was associated with a higher BMI Z-score (β=0.08, 95% CI=0.03-0.14), higher body fat (β=2.06, 95% CI=1.33-2.79) and an increased risk of obesity (OR=1.23, 95% CI=1.02-1.49). In a cross-sectional survey, frequency of fast food consumption was positively associated with body mass index (β=0.31, *P*=0.02), in adults.^16^ The association of fast foods and BMI was β=0.39 and 0.85 in high- and low-income in young and middle-aged women, respectively.^22^ In Singaporean adults, the risk of abdominal obesity was 1.24 (95% CI=1.03- 1.51) and 1.52 (95 % CI= 1.32- 1.77) in regular consumers and occasional consumers of fast food meals.^17^ In the Michigan Behavioral Risk Factor Survey, the adjusted-odds of obesity in adults consuming ≥3 times/week compared to <1 time/week fast food meals was 1.81 (95% CI=1.35-2.44).^18^ A significant association between fast food intake and BMI (β=0.104, *P*<0.01) as well as waist circumference (β=0.083, *P*<0.01) was observed among Iranian young adults.^19^ In Mediterranean adults, the association of fast food consumption with BMI was estimated to be β=1.76 (95% CI=0. 22, 3.3), and the risk of obesity increased by 129% in >1 time/week fast food consumers, compared to non-consumers.^2^ More interestingly, a health community survey in Michigan found a significant association between local concentrations of fast food outlets with BMI (β=3.21, *P*<0.001) and poor diet quality (β=2.67, *P*<0.008).^20^

Findings of a study on 23182 adolescents in Finland showed an strong association between fast-food outlet near school with breakfast skipping and undesirable eating habits; in this study, proximity of a fast-food outlet was associated with increased risk of overweight (OR=1.25, 95% CI=1.03-1.52).^23^ One study on the participants of National Health and Nutrition Examination Survey showed that fast food and full-service restaurant consumption, respectively, was associated with more energy, total fat and sodium intake as well as a decrease in daily intake of vitamin A, D, and K.^24^ Fast-food consumption was also significantly associated with higher intake of total energy (β=72.5, *P*=0.005), empty calories (β=0.40, *P*=0.006) and BMI (β=0.73, *P*=0.011), and lower healthy eating index score (β= -1·23, *P*=0·012), vegetables (β=-0·14, *P*=0·004), whole grains (β=-0.39, *P*=0·005), fiber (β= -0.83, *P*=0·002), magnesium (β=-6·99, *P*=0·019) and potassium intakes (β=-57.5, *P*=0·016).^25^

### Fast food consumption and dyslipidemia

Another cardiometabolic risk factor regarding fast food pattern highlighted in the literature is impaired metabolism of lipids and lipoproteins. In Coronary Artery Risk Development in Young Adults (CARDIA), participants who consumed ≥2.5 compared to <0.5 meal/week of fast food meals, had higher levels of serum triglycerides (117±3.6 mg/dl *vs.* 95±5.2 mg/dl), and lower high-density lipoprotein cholesterol (HDL-C) (52.0±0.7 mg/dl *vs.* 57.5±1.1 mg/dl), over 13 years of follow-up; moreover, longitudinal associations (β coefficient ± SE) of weekly fast food consumption with 13-year changes of triglycerides (TG), low-density lipoprotein cholesterol (LDL-C) and HDL-C were β=0.24±0.40, β=0.16±0.14, and β=0.08±0.06), respectively.^7^ A greater increase in 3-year changes of TG levels was found in Tehran Lipid and Glucose Study (TLGS) participants, who consumed more fast food meals at baseline (10.6% *vs.* 4.4% increase, in the fourth compared to first quartile of fast food intake); serum triglycerides to HDL-C ratio, an independent risk factor of cardiovascular disease, also increased in adults with higher compared to lower fast food intakes (3.7% *vs.* -5.5%, in the fourth compared to the first quartile).^8^

A cross-sectional analyses in TLGS study also indicated that fast food consumption (g/week) was significantly associated with serum TG (β=0.07, *P*<0.05), HDL-C (β= -0.05, *P*<0.05) and atherogenic index of plasma (β=0.06, *P*<0.05) only in middle-age adults; a higher prevalence of hypertriglyceridemia was also observed in the highest compared to the lowest tertile of fast foods (42.3 *vs.* 34.2%).^19^ Postprandial lipemia and lipid peroxidation increased after consumption of a fast food meal, compared to a healthy meal; triglyceride levels, malondialdehyde, and thiobarbituric acid reactive substances (TBARS) were significantly higher and HDL-C levels were significantly lower after fast food meal.^26^

### Fast food consumption and the risk of diabetes, metabolic syndrome and cardiovascular disease

The adverse effects of fast foods consumption on the development of metabolic abnormalities has been reported in several investigations. The associations of fast food consumption with the risk of insulin resistance, diabetes, metabolic syndrome and cardiovascular disease in cohort and cross-sectional studies were summarized in Table2. A 15-yrfollow-up of American women showed that higher fast food intake ≥ 2 times resulted in greater insulin resistance.^6^ In the CARDIA Study, participants in the 3^rd^ and 4^th^, compared to the first quartile category, of fast food intakes at baseline, had greater odds of metabolic syndrome after 13-yrof follow-up (OR= 1.9, 95% CI= 1.11-3.26 and OR= 2.14, 95% CI= 1.24-3.70, in 3^rd^ and 4^th^quartiles, respectively); homeostatic model assessment of insulin resistance (HOMA-IR) at final examination was also positively associated with fast food consumption at baseline (3.9±0.14 *vs.*0.3±0.18 in the highest compared to the lowest quartile of fast foods).A one-follow-up of adults showed that higher consumption of processed meat products was independently associated with the incidence of metabolic syndrome (OR= 2.5, 95% CI= 1.0-6.2).^30^

**Table2:** The association of fast food consumption with the risk of insulin resistance, diabetes, metabolic syndrome and cardiovascular disease in cohort and cross-sectional studies

Author	Design, study population and sample size	Findings
Pereira et al., 2005 ^(6)^	Fifteen-year follow-up of American women, n=3031	Consumption of fast foods ≥2 times/week increased the risk of insulin resistance.
Duffey et al., 2009 ^(7)^	Thirteen-year follow-up of adults participated in CARDIA study, n=36.43	Higher consumption of fast foods increased the risk of metabolic syndrome (OR= 1.9, 95% CI= 1.11-3.26) and (OR= 2.14, 95% CI= 1.24-3.70), in the 3rd and 4th quartiles, respectively). Higher insulin resistance index was observed in the highest compared to lowest quartile of fast foods (3.9±0.14 *vs.* 0.3±0.18, P<0.05).
Duffey et al., 2007 ^(14)^	One-year follow-up of adults, n=3394	Higher consumption of processed meat products was associated with the incidence of metabolic syndrome (OR= 2.5, 95% CI= 1.0-6.2).
Bahadoran et al., 2013 ^(8)^	Three-year follow-up of men and women participated in Tehran Lipid and Glucose Study, n=1476	The higher compared with the lower quartile of fast foods consumption increased the risk of metabolic syndrome by 85% (OR=1.85, 95% CI=1.17–2.95).
Odegaard et al., 2012 ^(29)^	Follow-up of Singaporean women, n= 43 176 for diabetes and n=52 584 for coronary heath disease mortality	Consumption of fast food ≥ 2 times/week increased the occurrence of type 2 diabetes (hazard ratio= 1.27, 95% CI= 1.03-1.54) and coronary heart disease mortality (hazard ratio = 1.56, 95% CI= 1.18-2.06).
Halton et al., 2006 ^(30)^	Twenty-year follow-up of women participated in Nurses' Health Study, n=84 555	Higher intake of French fries increased the risk of diabetes by 21% (OR=1.21, 95% CI=1.09-1.33).
Krishnan et al., 2010 ^(31)^	Ten-year follow-up of women participated in Black Women's Health Study, n=44 072	Higher intake of hamburgers and fried chicken (≥ 2 meals/week compared to none) increased incidence rate of type 2 diabetes by 1.40 (95% CI= 1.14, 1.73) and 1.68 (95% CI= 1.36, 2.08), respectively.
Alter et al., 2005 ^(9)^	Cross-sectional survey in Canada, n=380 regions	The higher compared to the lower accessibility to fastfood services increased the risk of mortality (OR= 2.52, 95% CI=1.54-4.13) and acute coronary hospitalizations (OR= 2.62, 95% CI=1.42-3.59).

The prospective approach of TLGS also showed that the risk of metabolic syndrome in the highest, compared with the lowest, quartile of fast foods increased by 85% (OR=1.85, 95% CI=1.17–2.95); in this study, the adverse effects of fast food consumption were more pronounced in younger adults (<30 yr), and participants who had greater waist to hip ratio, consumed less phytochemical-rich foods or had low-fiber diet (*P*<0.05).^8^ Non-alcoholic fatty liver disease, a hepatic feature of metabolic syndrome, could be a result of fast food consumption. In an intervention study, 4-wkconsumption of fast food meals (≥2meals/day) in healthy subjects increased serum levels of alanin aminotransferase (22.1±11 U/l to 69.3±76 U/l), insulin resistance index (0.89±0.42 to 1.6±0.83) and hepatic triglyceride content (1.1±1.9% to 2.8±4.8%) as well as body fat percent (20.1±9.8% to 23.8±8.6%).^31^

A prospective cohort of Singaporean women showed that consumption of fast food ≥2 times/wk increased the occurrence of type 2 diabetes (hazard ratio= 1.27, 95% CI= 1.03-1.54) and coronary heart disease mortality (hazard ratio = 1.56, 95% CI= 1.18-2.06).^27^

Increased consumption of burger, fried chicken meals, sausage and other processed meat products as well as French fries was associated with an increased risk of developing type 2 diabetes mellitus; a prospective study of 84,555 women in the Nurses' Health Study indicated that higher intake of French fries increased the 20-years risk of diabetes by 21% (OR=1.21, 95% CI=1.09-1.33).^28^ In Black Women's Health Study, the 10-year incidence rate of type 2 diabetes for higher intake of hamburgers and fried chicken (≥ 2 meals/week compared to none) was 1.40 (95% CI= 1.14, 1.73) and 1.68 (95% CI= 1.36, 2.08), respectively.^29^Meta-analysis of seven prospective cohorts found that higher consumption of processed meat increased the risk of type 2 diabetes by 19% (95% CI=1.11-1.27).^32^

More interestingly, rather than the consumption of fast foods, the rate of accessibility to fast food services has been reported as a risk factor for cardiovascular disease; risk-adjusted outcomes in regions with high compared to low accessibility to fast food services were greater for mortality (OR= 2.52, 95% CI=1.54-4.13) and acute coronary hospitalizations (OR= 2.62, 95% CI=1.42-3.59).^9^

## Discussion

This review provides further evidence warning us against the irreparable effects of fast food consumption on public health especially the increasing global burden of obesity and cardiovascular diseases. Frequent consumption of fast foods as well as out-of-home meals is a serious dietary risk factor for development of increasing trend of obesity and other related abnormalities. Higher consumption of fast foods has undesirable effects on dietary intake and overall diet quality, which leads to increased incidence of metabolic disorders including obesity, insulin resistance, type 2 diabetes as well as cardiovascular disorders.

Briefly, compared to non-consumers or <1 time/week, regular consumption of fast foods and out-of-home meals ≥1-3 times/week was associated with an 20-129%elevated risk of general and abdominal obesity.^9,15,17,18^ Increased risk of type 2 diabetes and metabolic syndrome in subjects with higher consumption of fast foods (mean ≥ 2 times/week) was reported 27-68% and 85-150%, respectively.^7,8,14,27-29^ Higher consumption of fast foods and higher exposure to multiple sources of accessible, cheap, energy-dense fast foods were also accompanied with a 56-162% increased risk of coronary heart disease mortality.^9,27^

Several possible mechanisms have been suggested to explain undesirable effects of fast foods on health status. A main factor describing the obesity-induced properties of fast foods is a high-energy dense modality. Most fast foods have an extremely high energy density, approximately 158 to 163 kcal per 100 gram of food; it also has been estimated that a fast food meal typically has an energy density twice the recommended a healthy diet and contains approximately 236 kcal/100 g.^33^

High energy density of foods may have adverse effects.^34^ In children, consumption of fast foods compared to non-consumers, led to greater intake of energy (>187 kcal/day), energy density (0.3 kcal/g), total fat (9g/d), carbohydrate (24 g/d), and added sugar (26g/d). ^35^ In adults, participants in the highest compared to the lowest quartile of fast food consumption also had more energy intake (>460 kcal/d), total fat (>2.5% of total energy), and cholesterol (>30 mg/d).^8^ The difference of calorie intake in fast food days, compared with non-fast food days was estimated to be within 400 kcal in overweight adolescents.^36^

High-fat content and inappropriate composition of fatty acids of fast foods is a main dietary risk for chronic disease. Mean total fat percent of beef hamburgers, chips, chicken hamburgers and hot dogs has been reported within 35.83±10.68%, 35.84±8.66%, 23.02±5.07%, and 34.02±13.49%, respectively; 28-52% of total fat was estimated as saturated fat.^37^

Large portion size, high amount of refined carbohydrates and added sugar, and high glycemic load are other characteristics that could explain the threatening properties of fast food meals.^38^ In some of the most popular fast foods, trans fats were up to 24g/serving.^4^Higher content of industrially produced trans fatty acids in fast foods is an important component leading to weight gain, abdominal fat accumulation, development of insulin resistance and cardiovascular events.^39^Furthermore, sodium content of fast foods is often higher than recommended amounts; in some common fast food meals, salt content was reported to range from 4.4 to 9.1 gr per meal;^40^ a high-salt diet besides increasing blood pressure also intensifies insulin resistance and metabolic syndrome features.^41^

Some of the mechanisms that could explain the metabolic outcomes of fast foods have been investigated in clinical and experimental studies. Postprandial adverse metabolic disorders including lipemia, oxidative stress and pro-inflammatory processes after eating a fast food meal observed in a human study are other possible explanations for cardiometabolic outcomes of fast foods.^26^ Compared to a healthy fast food meal (fiber rich sourdough rye bread, salad with vinegar, orange juice), a hamburger meal (hamburger, bacon, cola drink) was associated with higher postprandial serum levels of glucose and insulin.^42,43^

In animal models, fast food diet induced a phenotype of non-alcoholic fatty liver and steatohepatitis;^43^ in this study, fast food diet was accompanied with higher liver weight, serum concentration of aspartate aminotransferase, intra-acinar inflammation and development of steatosis. Higher expression of genes related to fibrosis, inflammation, endoplasmic reticulum stress, and lipoapoptosis also was induced by fast food diets; activated pathways of hepatocellular oxidative stress, profibrotic and pro-inflammatory pathways were observed.^44^ After a fast food meal, a severe decrease in plasma antioxidant vitamins including vitamin A, E and C, and zinc, as well as iron accumulation was observed in rats; decreased levels of superoxide dismutase, reduced gluthathione, and higher levels of thiobarbituric acid reactive substances, lipoprotein oxidation susceptibility, C reactive protein and tumor necrosis factor-alpha were also observed.^44^

This study was a narrative review and had some limitations, which should be considered; subjective nature of the search method, potential selection bias of the articles, probable missing of unpublished data and lack of using Preferred Reporting Items for Systematic Reviews and Meta-Analyses (PRISMA) to design and report of the study were the mains limitations. Further researches especially meta-analysis of current studies may provide a comprehensive and accurate picture for undesirable outcomes of fast food patterns. Moreover, further assessment of nutritional behaviors and social determinants of fast foods intakes among different populations could help to development of efficient health strategies.

## Conclusion

Considering to growing interest to out-of-home meals and high prevalence of fast food consumption, food policies with an emphasis on providing healthy foods, and making nutritional information at fast-food restaurants may help consumers to order more healthful or lower-calorie foods.

## Acknowledgments

The authors wish to thank Ms. N.Shiva for critical editing of English grammar and syntax.

## Conflict of interest

There is no conflict of interest.
